# A novel neutralizing antibody recognizing a conserved conformational epitope in PDCoV S1 protein and its therapeutic efficacy in piglets

**DOI:** 10.1128/jvi.02025-24

**Published:** 2025-01-22

**Authors:** Rui Chen, Guiping Zhou, Junpeng Yang, Rong Yuan, Ying Sun, Yixiao Liang, Rui Wu, Yiping Wen, Yiping Wang, Qin Zhao, Senyan Du, Qigui Yan, Sanjie Cao, Xiaobo Huang

**Affiliations:** 1 Research Center for Swine Diseases, College of Veterinary Medicine, Sichuan Agricultural University, Chengdu, China; 2 Chengdu Livestock and Poultry Genetic Resources Protection Center, Chengdu, China; 3 Sichuan Science-Observation Experimental Station for Veterinary Drugs and Veterinary Diagnostic Technology, Ministry of Agriculture, Chengdu, China; 4 Key Laboratory of Animal Disease and Human Health of Sichuan Province, Chengdu, China; 5 International Joint Research Center for Animal Disease Prevention and Control of Sichuan Province, Chengdu, China; Loyola University Chicago - Health Sciences Campus, Maywood, Illinois, USA

**Keywords:** porcine deltacoronavirus, neutralizing monoclonal antibody, conformational epitope, therapy experiments, piglet model

## Abstract

Porcine deltacoronavirus (PDCoV) is an enteric pathogen that burdens the global pig industry and is a public health concern. The development of effective antiviral therapies is necessary for the prevention and control of PDCoV, yet to date, there are few studies on the therapeutic potential of PDCoV-neutralizing antibodies. Here, we investigate the therapeutic potential of a novel monoclonal antibody (mAb 4A6) which targets the PDCoV S1 protein and effectively neutralizes PDCoV, both pre- and post-attachment on cells, with IC50 values of 0.537 and 8.487 µg/mL, respectively. A phage-display peptide library was used to determine the epitope recognized by mAb 4A6, and two mimotopes, QYPVSYA (P1) and FPHWPTI (P2), were identified. KLH-P1 reacted with PDCoV-positive sera but failed to induce PDCoV-specific IgG and neutralizing antibodies in mice, suggesting P1 does not fully mimic the conformational epitope. Molecular docking and alanine scanning mutagenesis revealed that S461, P462, T463, E465, and Y467 on the S protein are essential for mAb 4A6 binding. Antibody therapy experiments in PDCoV-infected piglets showed that administering mAb 4A6 once or twice could delay the onset of diarrhea symptoms, reduce the severity of diarrhea, and decrease virus shedding. Taken together, our findings demonstrate that mAb 4A6 holds promise as a treatment against PDCoV, and the amino acids recognized by mAb 4A6 will be valuable for developing novel epitope-based vaccines or antiviral drugs.

**IMPORTANCE:**

Porcine deltacoronavirus (PDCoV) is a novel swine enteropathogenic coronavirus that poses a potential threat to public health. Developing effective antiviral therapies is crucial for its prevention and control. Here, we demonstrated that mAb 4A6 shows promise as a treatment against PDCoV. Antibody therapy experiments conducted on PDCoV-infected piglets revealed that administering mAb 4A6 once or twice could delay the onset of diarrhea symptoms, reduce the severity of diarrhea, and decrease virus shedding. Furthermore, we characterized the conformational epitope (S461, P462, T463, E465, and Y467) recognized by mAb 4A6 through an integrated approach involving phage display peptide library, molecular docking, and alanine scanning mutagenesis. More importantly, mAb 4A6 exhibits a broad-spectrum neutralizing activity against different PDCoV strains. These findings indicate that mAb 4A6 has promising therapeutic value for PDCoV-infected piglets, and the identification of mAb 4A6 recognized epitope may provide a new idea for the identification of conformational epitopes.

## INTRODUCTION

Coronaviruses (CoVs) are a diverse group of enveloped, single-stranded RNA viruses with the ability to infect both human and animal populations. One prominent example is severe acute respiratory syndrome coronavirus 2 (SARS-CoV-2), which was responsible for the COVID-19 pandemic (1). The virus is believed to have originated from bat coronaviruses (2–4) and to have jumped to humans through an intermediate animal host (5), although the exact host has yet to be definitively identified. Similarly, in 2012, Middle East respiratory syndrome CoV (MERS-CoV) emerged in Saudi Arabia (6), and although it has not established sustained human infections, it is recurrently re-emerged from its reservoir, the dromedary camel (7, 8). These occurrences serve as poignant illustrations of CoVs’ ability to jump species and the ongoing risk to human and animal health.

CoVs are classified into four genera: *Alphacoronavirus*, *Betacoronavirus*, *Gammacoronavirus*, and *Deltacoronavirus*. The first two genera cause infections only in mammals, while birds and mammals are commonly infected by *Gamma*- and *Delta-coronaviruses* (9). Porcine deltacoronavirus (PDCoV) was first discovered in fecal samples of pigs in Hong Kong in 2012, though its origin remains unclear (9). Since its initial detection, PDCoV has been widely reported in the Americas and Asia and has become a common pathogen in pig populations (10–17). PDCoV infects intestinal epithelial cells and causes serious diarrhea, vomiting, dehydration, and mortality in suckling piglets. Symptomatic PDCoV infection has also been observed in calves, chickens, poults, and mice in experimental settings (18–21), an indication of the wide host range potential. In 2021, PDCoV was detected in plasma samples from three children with acute febrile illness (22). This discovery raises public health concerns about PDCoV transmission from swine to humans.

The initial step in CoV infection involves the binding of the viral spike (S) protein to a receptor on the host cell surface (23). The S protein is also the primary target of the humoral immune response during infection. Buchhol et al. (24) report that a single intranasal administration of parainfluenza virus type 3 vector expressing the SARS-CoV S protein produced high levels of neutralizing antibodies, comparable to those from SARS-CoV infection. Because of its significant role in infection, the S protein has been the target for the development of vaccines and therapeutics.

Neutralizing antibodies are important components of the humoral immune system against viral infection, and their potency as therapeutic agents is underscored by their pivotal role in countering CoVs. A large majority of neutralizing monoclonal antibodies (mAbs) isolated from COVID-19 patients were found to target the S1 protein (25). Most of these neutralizing mAbs target the epitope of the receptor-binding domain (RBD) that intersects with the RBD-human angiotensin-converting enzyme 2 (hACE2) interface, which directly obstructs the hACE2 binding (26, 27). Of course, numerous neutralizing mAbs also have been found targeting the S protein of SARS-CoV, MERS-CoV, and various porcine CoVs (28–33). For example, numerous neutralizing mAbs targeting the PEDV S protein have been reported (31, 33, 34). Some of these antibodies, such as single-chain fragment variable antibodies (PZZ 21, PZZ 24, and PZZ 35), have shown promise for the prevention and treatment of PEDV infection in pigs (35). However, despite the zoonotic potential of PDCoV, the precise epitopes and functional attributes of PDCoV-neutralizing antibodies remain less well explored.

**Fig 1:**
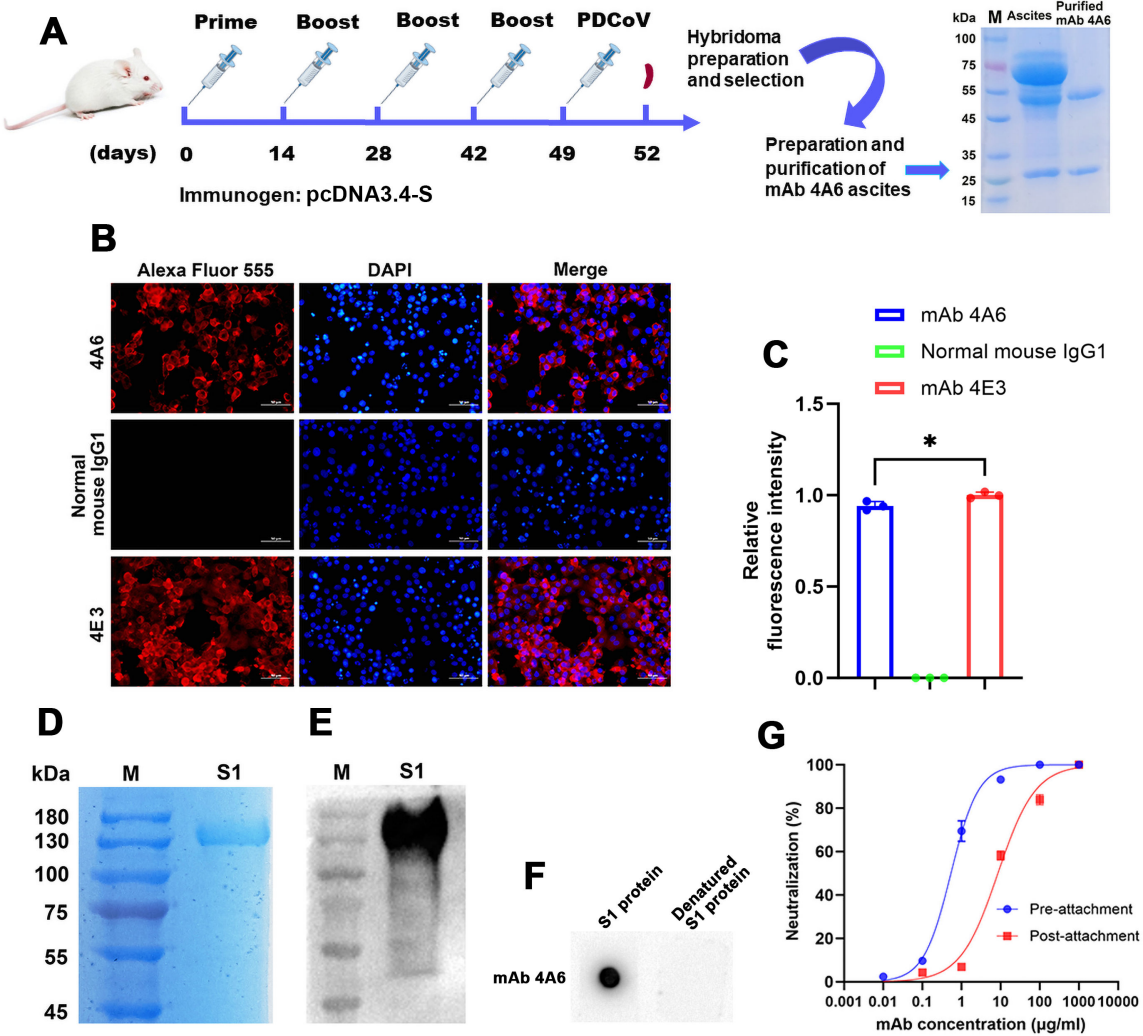
Characterization of mAb 4A6. (**A**) Schematic diagram of the screening and purification of mAb 4A6. (**B**) Indirect immunofluorescence analysis (IFA) of the mAb 4A6 in PDCoV-infected ST cells. (**C**) Quantitative analysis of relative fluorescence intensity using *ImageJ*. Relative fluorescence intensity after mAb 4A6 staining was normalized against relative fluorescence intensity after mAb 4E3 staining. Data were analyzed using three pictures for each mAb and shown as mean ± SD. *P < 0.05; one-way ANOVA. (**D**) Coomassie of purified PDCoV S1. (**E**) PDCoV S1 is specifically recognized on a western blot using PDCoV mAb 4E3. (**F**) Characterization of epitope recognized by mAb 4A6 using dot blot. Purified PDCoV S1 protein was mixed with 6× protein-loading buffer with DTT (TransGen Biotech, Beijing, China) then heated for 10 min at 95°C then subjected to dot blot with the mAb 4A6. (**G**) mAb 4A6 was tested for neutralizing activity against PDCoV before and after virus adsorption using a plaque reduction neutralization test (PRNT). The IC50 values were calculated using *GraphPad Prism 8.0*.

In preliminary experiments (unpublished), we produced PDCoV-neutralizing mAbs using BALB/C mice immunized with a eukaryotic plasmid expressing the full-length PDCoV S protein. Of the mAbs obtained, mAb 4A6, which recognizes a conformational epitope on the S protein, had the greatest PDCoV-neutralizing capacity (Fig. 1A). Here, we evaluated the neutralization efficiency of mAb 4A6 in pre- and post-PDCoV-infected cells, determined the essential amino acids in the S protein needed for 4A6 binding, and assessed the therapeutic efficacy of mAb 4A6 in PDCoV-infected piglets. Our findings provide evidence that mAb 4A6 has potential as a therapeutic drug against PDCoV variants in humans and animals; identification of the essential amino acids recognized by mAb 4A6 will also be valuable for the development of novel epitope-based vaccines or antiviral drugs.

## RESULTS

### Identification of reactivity and neutralizing ability of mAb 4A6

Figure 1A illustrates the immunization of BALB/c mice with pcDNA3.4-S (containing the full-length S gene) and subsequent purification of mAb 4A6 done in our previous study. Here, we show by IFA that mAb 4A6 reacts with PDCoV-infected ST cells (Fig. 1B). However, the observed fluorescence intensities were comparatively weaker than those elicited by mAb 4E3 (Fig. 1C). Figure 1D and E show the purification of the PDCoV S1 protein using Coomassie and western blot. Dot-blot result showed that denatured PDCoV S1 protein was not recognized by mAb 4A6, indicating that mAb 4A6 recognizes a conformational epitope (Fig. 1F). Subsequently, we investigated whether mAb 4A6 could inhibit the invasion of PDCoV at both pre- and post-attachment steps (Fig. 1G). As the data indicated, mAb 4A6 inhibited the entry of PDCoV before and after virus adsorption (IC50 values: 0.537 and 8.487 µg/mL, respectively).

### Identification and immunogenicity analysis of epitope recognized by mAb 4A6

**Fig 2:**
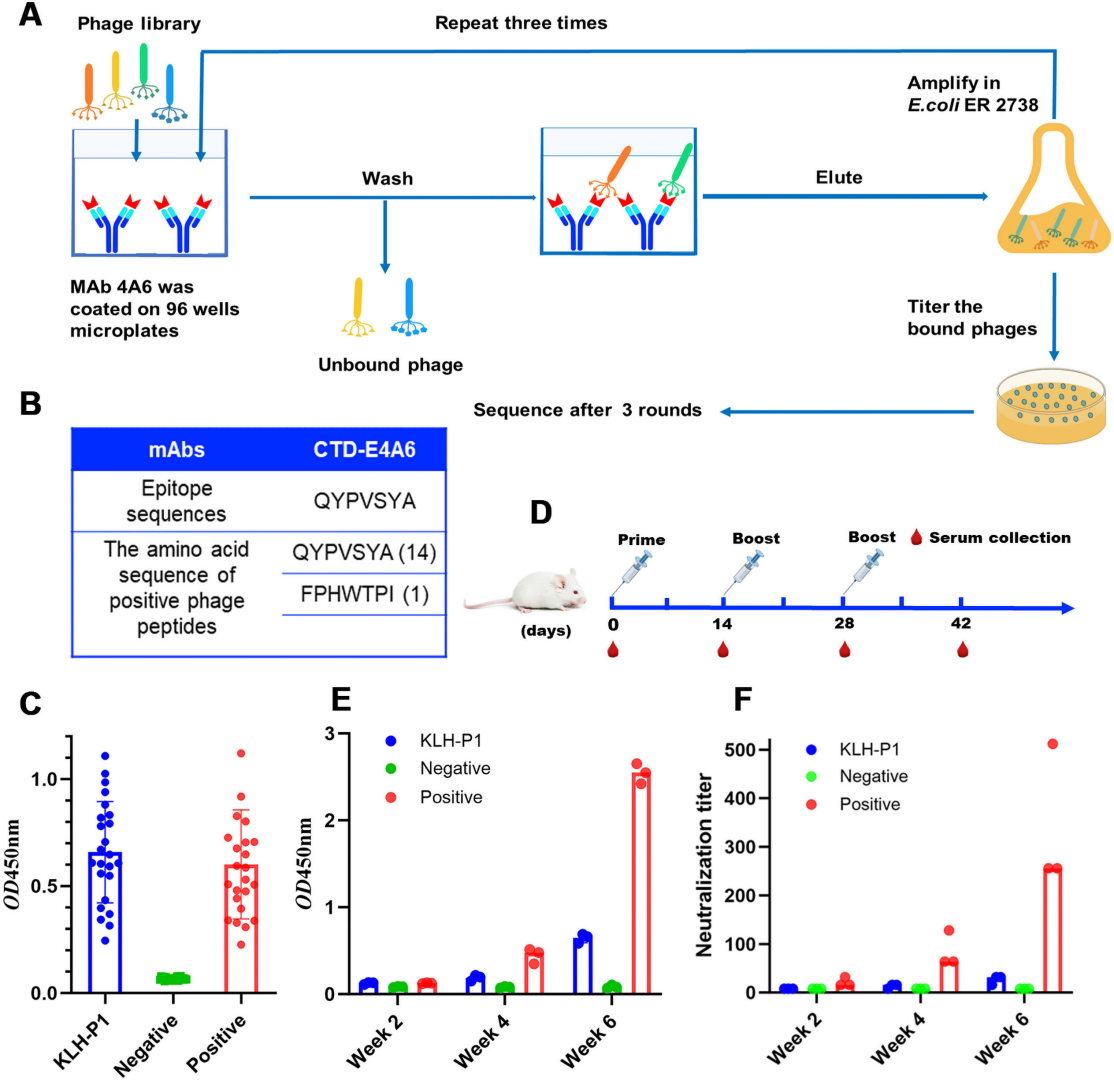
Identification and immunogenicity analysis of the epitope recognized by mAb 4A6. (**A**) The workflow for panning the Ph.D.-7 phage display peptide library using mAb 4A6. (**B**) The peptides identified from mAb 4A6 reacting phage clones. QYPVSYA was commercially synthesized and conjugated to the carrier KLH. (**C**) Antibody responses against the mimotope were assessed using ELISA with 24 PDCoV-positive pig sera. ELISA plates were coated with epitope-conjugated KLH proteins, and the serum was diluted 1:200 for use in the ELISA. (**D**) Diagram illustrating the experimental schedule. BALB/C mice were immunized according to the immunization procedure. Three mice per group were vaccinated with different epitope-conjugated KLH proteins on days 0, 14, 28, and 42. Serum was collected periodically. (**E**) Levels of anti-P1 IgG, as measured by ELISA, in immunized and control mice. (**F**) PDCoV-neutralizing titers. All experiments incorporated positive and negative controls, with the epitope aa 280–288 recognized by mAb 4E3 serving as the positive control and the Pan HLA DR-binding epitope (PADRE) as the negative control. Both of these epitopes were conjugated to KLH.

To identify the epitope recognized by mAb 4A6, a commercial Ph.D.-7 Phage Display Peptide Library was screened by using mAb 4A6 with a workflow as depicted in Fig. 2A. After three rounds of affinity biopanning, 15 phage clones were randomly picked for DNA sequencing. Of these, 14 had an identical peptide with the deduced amino acid sequence “QYPVSYA” denoted as P1, and one clone had a peptide with a deduced amino acid sequence “FPHWPTI” denoted as P2 (Fig. 2B). Sequence alignment results showed that neither P1 nor P2 corresponded with the amino acid sequence of PDCoV S. Consequently, the P1 and P2 can only function as mimotopes. To assess whether the P1 mimotope is recognized by PDCoV-positive serum, the P1 peptide, with a cysteine residue at the N terminus, was synthesized and conjugated to keyhole limpet hemocyanin (KLH). The reactivity of the synthesized peptide with 24 PDCoV-positive pig sera was determined by indirect ELISA. The results showed that P1 was recognized by all 24 PDCoV-positive pig sera (Fig. 2C).

To evaluate the immunogenicity of KLH-P1, BALB/c mice were immunized with KLH-P1 with a workflow as depicted in Fig. 2D. The epitope aa 280–288, recognized by mAb 4E3 (36), served as the positive control, while the Pan HLA DR-binding epitope (PADRE) was the negative control. Sera were collected at 2-week intervals after the primary inoculation, and PDCoV-specific antibodies were detected using ELISA. The results showed that KLH-P1 induced only a low level of PDCoV-IgG antibodies after the third inoculation (Fig. 2E). PDCoV neutralization was detected 2 weeks after mice were initially inoculated. Sera from the KLH-P1 inoculated mice had no significant neutralizing activity until week 6, and then only at a low-level (>1:16) PDCoV-neutralizing antibody titers (Fig. 2F).

### Identification of the essential residues involved in mAb 4A6 binding

**Fig 3:**
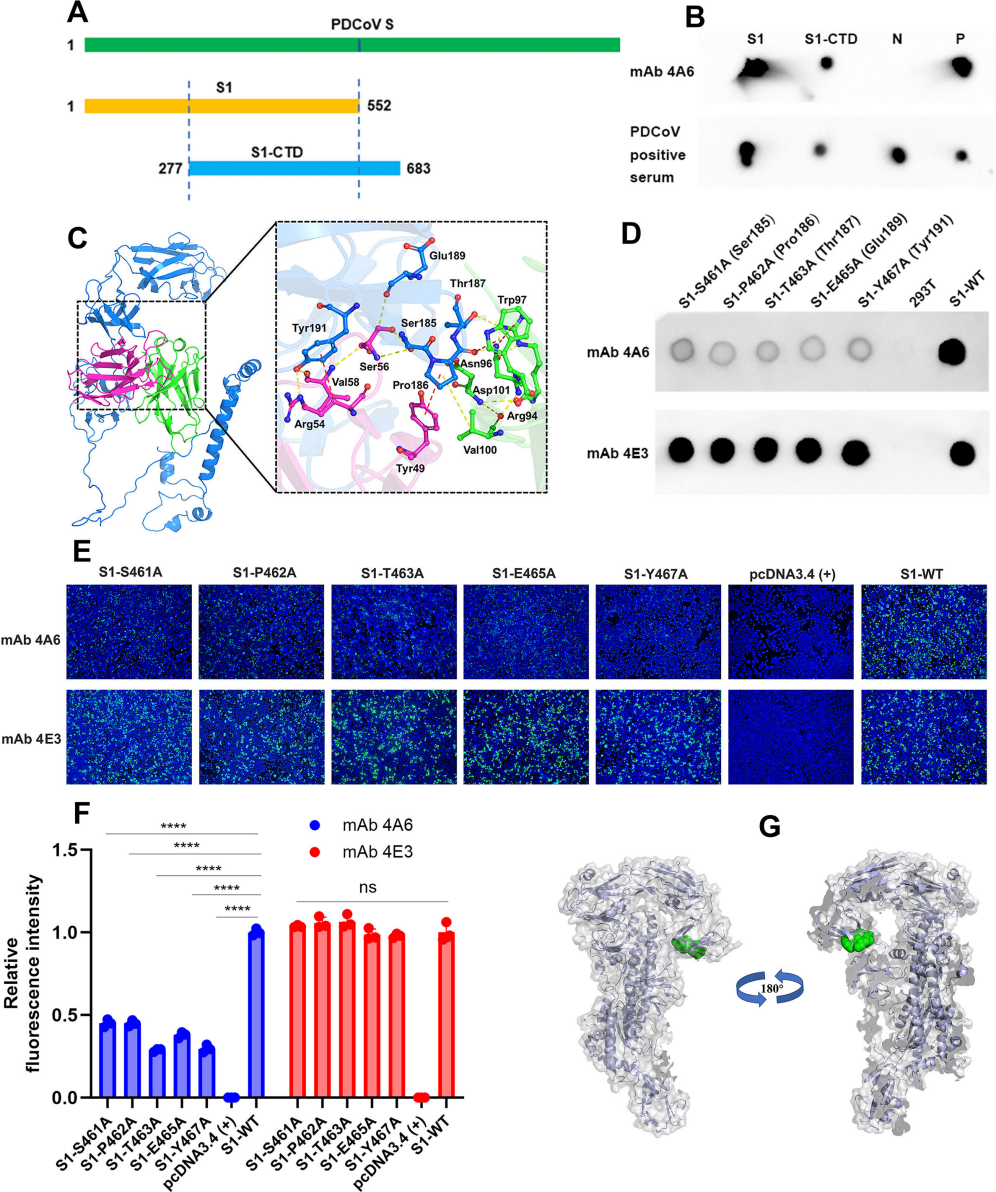
Identification of the essential amino acid involved in mAb 4A6 binding. (**A**) Schematic of the location and length of the PDCoV S1 (aa 1–552) and S1-CTD (aa 277–683). (**B**) Binding results of mAb 4A6 with PDCoV S1 and S1-CTD proteins using dot blot assays. “N” meant the purified PDCoV N protein, which serves as a negative control; “P” meant the purified PDCoV virions, which serves as a positive control. (**C**) Amino acids predicted to be involved in mAb 4A6 binding. The S1-CTD protein is colored blue, while the light and heavy chains of mAb 4A6 are colored pink and green, respectively. PDCoV S1 constructs containing the indicated mutation were transfected into cells and analyzed by (**D**) dot blotting and (**E**) IFA. (**F**) Quantitative analysis of the relative fluorescence intensity in (**E**). The relative fluorescence intensity of the five single-point mutation PDCoV S1 proteins was normalized to the relative fluorescence intensity of the wild-type S1 protein. Three different pictures for each replicate were analyzed using the *Image J*, and data were represented as mean ± SD. ****P < 0.0001; two-way ANOVA. (**G**) Spatial localization of the essential amino acid involved in mAb 4A6 binding. The 3D model of the PDCoV S protein (PDB ID: 6b7n) was obtained from Protein Data Bank (PDB), and the figures were generated using the *PyMol* molecular visualization system. The essential amino acid involved in mAb 4A6 binding is in green.

To begin to identify the precise region of the S protein recognized by mAb 4A6, we performed a dot blot assay on purified S1 and S1-CTD and found that mAb 4A6 recognized both proteins, suggesting the binding epitope is within the aa 277–552 region (Fig. 3A and B). The binding region was analyzed using *MOE* software, and the interactions of modeled results were analyzed by *PyMOL*. Five residues that are likely involved in binding are colored blue in Fig. 3C. Then, five single-point mutations (S1-S461A, S1-P462A, S1-T463A, S1-E465A, and S1-Y467A), designed according to their interaction characteristics, were introduced into S1 constructs, and the binding of mAb 4A6 was detected using dot blot assay. As shown in Fig. 3D, each of the mutations nearly abolished the binding of mAb 4A6. The findings from the IFA and the subsequent quantification of relative fluorescence intensity also demonstrated that each mutation significantly disrupted the interaction between the mAb 4A6 and the S1 protein (Fig. 3E and F). These results indicate that these five amino acids (S461, P462, T463, E465, and Y467) are crucial for the binding of mAb 4A6.

### Sequence alignment of the essential amino acid residues recognized mAb 4A6 among different CoV strains

**Fig 4:**
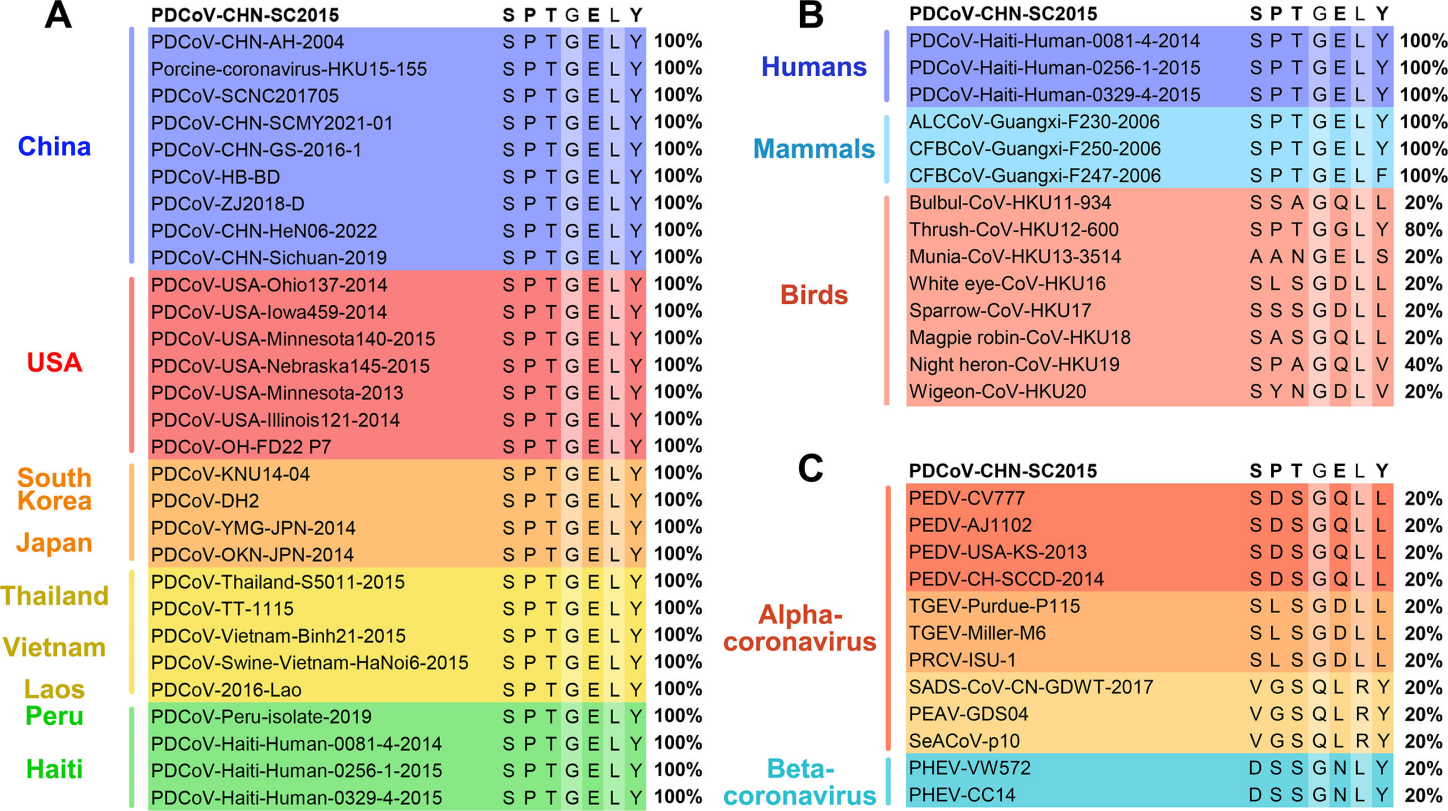
Comparison of the essential residues involved in mAb 4A6 binding among CoV strains. (**A**) Alignment against 25 reference strains of PDCoV. (**B**) Alignment against delta-coronavirus strains from 11 species. (**C**) Alignment against 12 strains of alpha- and beta-porcine CoVs. *MEGA-X* and *DNASTAR* were used to analyze the levels of homology. Different groups are marked with different colors.

To determine the level of conservation of the five amino acid residues, 25 reference PDCoV strains from nine countries were selected for alignment analysis (Fig. 4A). The results revealed that the five residues are completely conserved among PDCoV strains, with amino acid identity of 100%. Delta-CoV strains from 11 species (humans, mammals, and birds) were also selected for sequence alignment (Fig. 4B). The results showed that PDCoV CHN-2015 and all the mammalian strains (human, Chinese ferret-badger coronavirus [CFBCoV], and Asian leopard cat coronavirus [ALCCoV]) share 100% sequence identity at all five residues. Sequence identity with the avian strains ranges from 20% to 80%. Of note, thrush-CoV shares 80% sequence identity at S461, P462, T463, E465, and Y467, while other avian strains exhibit considerably lower levels of sequence identity. Figure 4C shows that the sequence identity of these five residues between PDCoV CHN-2015 and other pig-origin alpha-CoVs (PEDV, TGEV, SADS-CoV, and PRCV) and beta-coronavirus strains (PHEV) is 20%.

### Assessment of the broad-spectrum efficacy of 4A6 against various PDCoV strains

**Fig 5:**
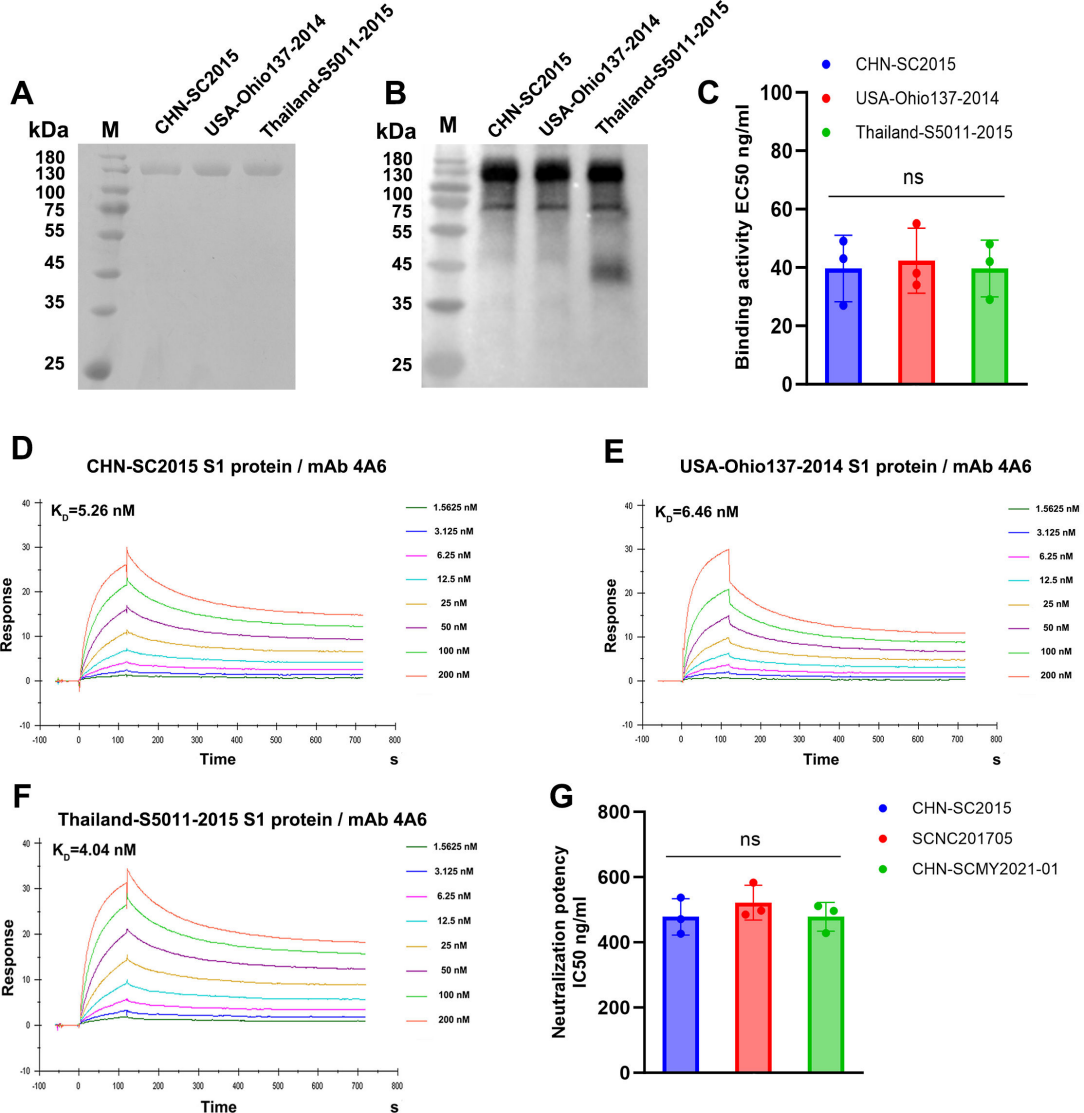
Assessment of the broad-spectrum efficacy of 4A6 against various PDCoV strains. (**A**) The Coomassie staining of purified S1 proteins of the PDCoV strains CHN-SC2015, USA-Ohio137-2014, and Thailand-S5011-2015. (**B**) The expression of S1 proteins was confirmed using western blot analysis with PDCoV-positive serum. Purified S1 protein samples were separated by SDS-PAGE, subsequently transferred to a PVDF membrane and immunoblotted with PDCoV-positive serum. (**C**) Reactivity as measured by ELISA of mAb 4A6 to purified S1 proteins. Purified S1 proteins at 1 µg/mL were used to coat plates overnight at 4°C, and mAb 4A6 was serially diluted in PBS and assessed for binding affinity. Half-maximal binding titer (EC50) was calculated by curve fitting in *GraphPad Prism 8.0*. The data are represented as the mean  ±  SD from three independent experiments. Statistical significance was determined by one-way ANOVA. (**D–F**) The binding kinetics between mAb 4A6 and PDCoV S1 proteins using SPR. The KD values were calculated from sensograms using eight mAb 4A6 concentrations (200, 100, 50, 25, 12.5, 6.25, 3.125, and 1.5625 nM). (**G**) mAb 4A6 was tested for neutralizing activity against PDCoV stains CHN-SC2015, SCNC201705, and CHN-SCMY2021-01 using a plaque reduction neutralization test (PRNT). Each mAb was diluted 10-fold from 100 µg and tested. The IC50 values were calculated using *GraphPad Prism 8.0*. The data are represented as the mean  ±  SD from three independent experiments. Statistical significance was determined by one-way ANOVA.

In order to evaluate the binding ability of mAb 4A6 to the S1 protein across various PDCoV strains, the S1 proteins from the Chinese isolate (CHN-SC2015), the American isolate (USA-Ohio137-2014), and the Thai isolate (Thai-S5011-2015) were successfully expressed and purified (Fig. 5A and B). The results of the indirect ELISA assay indicated that mAb 4A6 exhibited similar reactivity toward the S1 protein of PDCoV strains CHN-SC2015, USA-Ohio137-2014, and Thai-S5011-2015, with mean EC50 values of 39.67, 42.33, and 39.67 ng/mL, respectively (Fig. 5C). Subsequently, the binding rate constant of mAb 4A6 to the three PDCoV S1 proteins was determined using surface plasmon resonance (SPR). As shown in Fig. 5D through F, mAb 4A6 exhibited nanomolar affinity for all three PDCoV S1 proteins, with affinity constants (KD) of 5.26, 6.46, and 4.04 nM, respectively. Importantly, we observed that mAb 4A6 effectively neutralized three distinct PDCoV isolates stored in our laboratory (Fig. 5G). These findings indicate that mAb 4A6 possesses broad neutralizing activity.

### mAb 4A6 therapy can delay the onset of diarrhea in PDCoV-infected pigs

**Fig 6:**
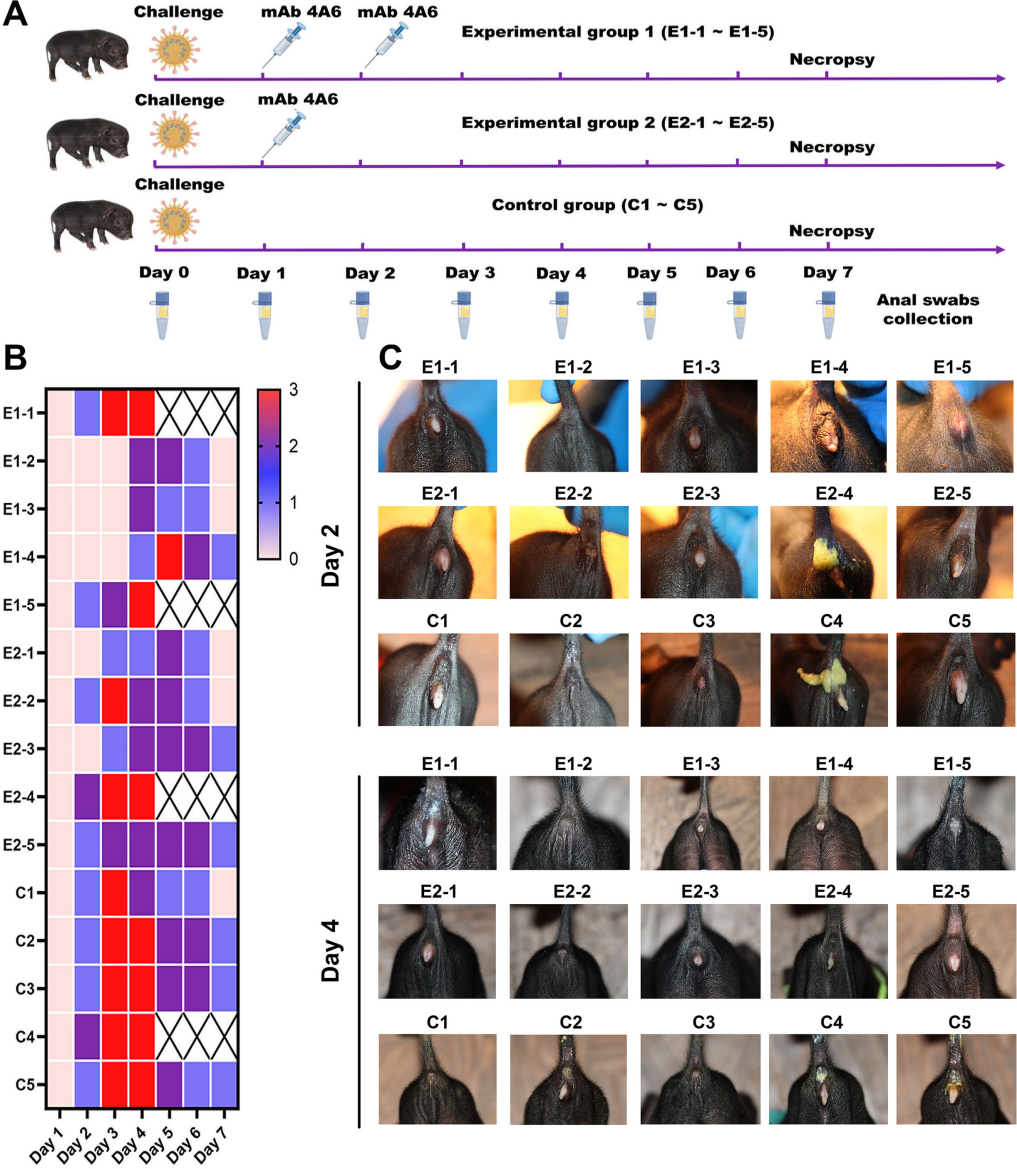
Diarrhea in PDCoV-infected piglets. (**A**) Experimental design for accessing mAb 4A6 as a therapeutic. mAb 4A6 (3 mg/kg) was intramuscularly injected into the pigs after PDCoV infection. Diarrhea in pigs was assessed daily. (**B**) Fecal consistency was scored for each piglet as follows: 0 = normal (solid); 1 = pasty; 2 = semiliquid diarrhea; and 3 = liquid. (**C**) Diarrheal symptoms of pigs on days 2 and 4 following PDCoV infection.

The effectiveness of mAb 4A6 as a therapeutic agent against PDCoV *in vivo* was evaluated; the workflow is depicted in Fig. 6A. The diarrhea symptoms of each pig were recorded daily after PDCoV challenge. As shown in Fig. 6B and C, all pigs in the control group exhibited diarrhea on the second day following the viral challenge, whereas only one and two pigs in experimental groups 1 and 2, respectively, displayed signs of diarrhea. Furthermore, therapeutic use of mAb 4A6 resulted in a decrease in the severity of diarrhea, with a majority of pigs in the experimental groups exhibiting no instances of liquid stools. These findings suggested that therapy with mAb 4A6 can delay the onset of pig diarrhea symptoms and mitigate the severity of the condition.

### mAb 4A6 showed partial therapeutic efficacy in PDCoV-infected piglets

**Fig 7:**
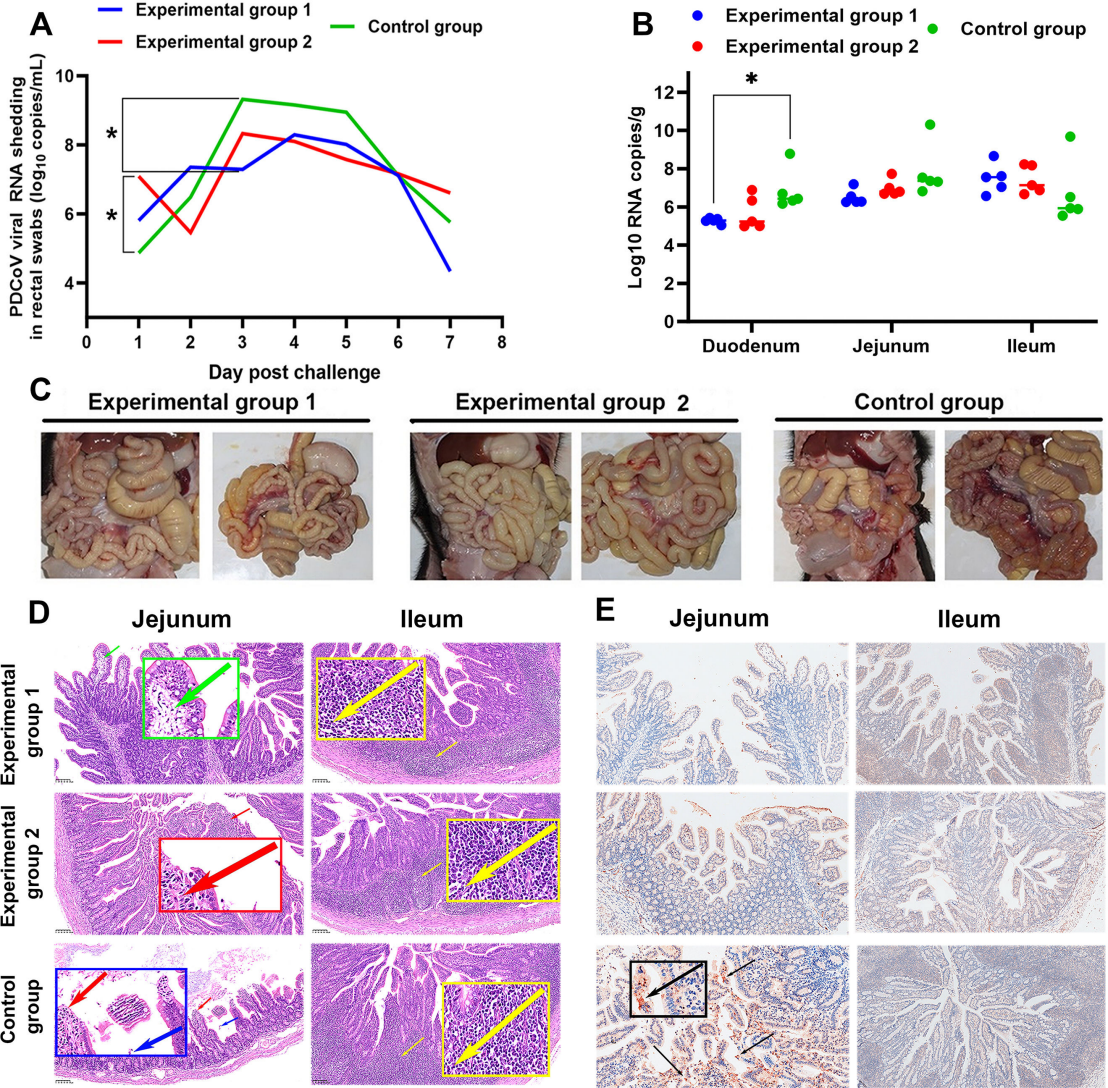
mAb 4A6 showed partial therapeutic efficacy in PDCoV-infected piglets. (**A**) Viral shedding from challenged piglets. Fecal swabs were collected from each piglet once a day, and viral load was quantitated by qRT-PCR targeting the PDCoV *M* gene. Data presented as the means ± SD of five piglets per group; *P < 0.05; two-way ANOVA. (**B**) Distribution of viral load in challenged piglets. Viral load was quantitated by qRT-PCR targeting the PDCoV *M* gene. Data presented as the means ± SD of five piglets per group; *P < 0.05; two-way ANOVA. (**C**) Gross lesions in challenged piglets. (**D**) Histological analysis of jejunum and ileum sections from challenged piglets. Arrows indicate typical histological lesions in these different tissues. Green arrows: loose cytoplasm; red arrows: exposed lamina propria; blue arrows: exfoliation of the small-intestine epithelial; yellow arrows: lymph follicle. Scale bars are shown in each picture (magnification, 200×). (**E**) Immunohistochemistry (IHC) staining of virus in the jejunum and ileum from groups E1, E2, and control piglets (magnification, 200×).

The kinetics of virus shedding are shown in Fig. 7A. Control group piglets began to shed virus in the feces at 1 day post-challenge (dpc), with levels increasing until day 3, with the greatest fecal viral load (10^9.16^ copies of PDCoV RNA per milliliter of fecal swab supernatant). Virus shedding in experimental group 1 increased more gradually than in the controls reaching a peak load on day 4 (10^8.29^ copies). Compared with the control group, the piglets in experimental group 1 had significant differences (*P* < 0.05) in virus shedding at 3 dpc. Virus shedding in experimental group 2 initially decreased, then rose to peak load (10^8.32^ copies) at day 3. Using qRT-PCR, we quantitated the viral load in the duodenums, jejunums, and ileums of each piglet (Fig. 7B). Compared to the control group, the duodenums of E1 group piglets had significantly lower levels of PDCoV RNA.

At 7 dpc, all piglets were euthanized and necropsied. In all groups, the small intestines were gas-distended and transparent, with thin-walls and mesenteric hemorrhage and lymph node enlargement. Comparatively, bleeding and congestion in the intestinal mucosa and mesenteric lymph nodes were more severe in the control group (Fig. 7C). Hematoxylin-eosin (HE) staining showed no significant differences in pathology between the experimental and control groups, except that the epithelial cells in the jejunum of the control group were significantly exfoliated (Fig. 7D). Immunohistochemistry (IHC) staining showed a higher viral load in jejunal epithelial cells of the control group piglets than in the two experimental group piglets (Fig. 7E).

## DISCUSSION

The outbreak of SARS-CoV-2 has drawn heightened attention to CoVs. Although many vaccines have been approved, therapeutic neutralizing mAbs against CoVs remains worthy of attention. The systematic characterization of epitopes targeted by CoV-neutralizing mAbs is valuable in the effort of CoV prevention and control. Currently, there are several anti-SARS-CoV-2 mAbs authorized for treatment, including the combinations of bamlanivimab/etesevimab, casirivimab/imdevimab, and sotrovimab/bebtelovimab (37). Neutralizing mAbs targeting animal CoVs are being developed rapidly. For instance, an anti-PEDV mAb, designated PC10, was isolated from porcine B cells and specifically recognizes the native PEDV S protein. This mAb robustly neutralizes both PEDV G1 and G2 strains, making it a promising candidate for passive protection (31).

Despite its recognition as a potential zoonotic pathogen after it was detected in the plasma from three children (22), the precise epitopes and functional attributes of PDCoV-neutralizing mAbs remain thinly explored. In our previous study (36), we showed that three regions of PDCoV S protein, S1-NTD (aa 50–286), S1-CTD (aa 278–616), and S2 (aa 601–1087), induce neutralizing antibody responses, with S1-CTD being the major immunodominant domain. Subsequently, mAbs targeting the S1-CTD protein were developed, of these, mAb 4E3 recognizes the 280-FYSDPKSAV-288 epitope in the SD2ʹ region. Du et al. (38) successfully obtained four neutralizing mAbs. Among these, three mAbs (42H3, 46E6, and 67B12) exhibited binding affinity to the S1B domain (also referred to as the C-terminal domain, CTD), while the remaining mAb (22C10) targeted the S1A domain (also referred to as the N-terminal domain, NTD) of the PDCoV S protein. Here, we identify a novel PDCoV neutralizing mAb (4A6), which recognizes an epitope situated outside the S1A domain (the N-terminal domain, NTD) and S1B domain (the C-terminal domain, CTD) of the PDCoV S protein.

We found that mAb 4A6 was 10-fold more effective at blocking PDCoV infection when ST cells were treated prior to viral attachment than when treated post-attachment (Fig. 1G). When the S1 subunit of CoV binds to the cell receptor, the non-covalent bond between the S1 and S2 subunits is broken due to the proteolytic cleavage of an S, and the S2 subunit forms a six-helix bundle fusion core by conformational change and activates further fusion processes (39). Blocking the interaction between the S1 subunit and the cell receptor is the main mechanism of most S1-directed neutralizing mAbs (25, 40). As shown by dot blot (Fig. 3A and B), mAb 4A6 specifically binds to aa 277–552 of the PDCoV S protein, which overlaps with the receptor-binding domain (aa 277–422) of the S protein. Therefore, mAb 4A6 presumably disrupts the interaction between the PDCoV S1 subunit and cellular receptors, thereby inhibiting the conformational changes of the S protein and consequently impeding PDCoV infection of host cells.

Linear antigen epitopes are commonly identified using peptide scanning technology. Using this method, we previously identified a linear epitope (280-FYSDPKSAV-288) on the PDCoV S protein (36) and another (309-KPKQKKPK-317) on the N protein (41). This methodology is not suitable for identifying conformational epitopes. The gold standard for identifying conformational epitopes is through structural biology methods such as X-ray crystallography and cryogenic-electron microscopy (42); these techniques, however, are time-consuming and costly. Phage-display peptide libraries have become a well-established method for identifying conformational antigen epitopes (43, 44). Hence, in this study, we utilized phage-display peptide libraries to identify the mAb 4A6 recognized epitope. Two motifs QYPVSYA (P1) and FPHWPTI (P2) were identified. However, they did not align with the amino acid sequence of the PDCoV S protein. As a result, P1 and P2 can only serve as mimotopes (Fig. 2B). Mimotopes are linear peptides that can fold themselves into short structural elements, mimicking the target epitope of a given antibody so closely that they are recognized by this antibody (45). They can block the interactions between an antibody and its real target and evoke similar responses when used as immunogens/vaccines, even if they do not share amino acid sequences with the recognized target. Mimotopes can also mimic conformational epitopes (46, 47). Given that the foreign amino acid sequence present in the majority of positive phage clones is QYPVSYA, we then evaluated the reactogenicity and immunogenicity of P1. As shown in Fig. 2E and F, KLH-P1 was recognized by 24 PDCoV-positive pig sera, but it did not elicit significant levels of PDCoV-specific IgG and neutralizing antibodies in mice, indicating that P1 does not fully mimic the conformational epitope recognized by mAb 4A6.

Shi et al. (30) used molecular docking to demonstrate that mAb 5H10 specifically targets motif E1 (147–167 amino acids) of the HCoV-229E S protein, and by alanine scanning, they identified residue F159 as essential for the binding of mAb 5H10. Therefore, we decided to use the same techniques and found that mAb 4A6 specifically binds to aa 277–552 of S protein (Fig. 3A and B). Consequently, the structures of the S1 (aa 1–552) and S1-CTD (aa 277–683) protein could both be used for molecular docking analysis, though we selected S1-CTD. The analysis revealed that the S protein residues S461, P462, T463, E465, and Y467 were likely to interact with mAb 4A6. Alanine scanning revealed that all the residues were necessary for mAb 4A6 binding (Fig. 3D and E). Of note, the mimotope P1 (QYPVSYA) shares three amino acids (S, P, and Y) with the key amino acids recognized by mAb 4A6, while P2 (FPHWPTI) shares two amino acids (*P* and T). This suggests that S461, P462, T463, E465, and Y467 may form the conformational epitope recognized by mAb 4A6. P1 does not constitute a complete epitope, which explains why P1-KLH conjugates did not induce significant PDCoV-specific IgG and neutralizing antibodies in mice. These results also indicated that molecular docking in combination with alanine scanning offers clear advantages in precisely identifying conformational epitopes and critical amino acids recognized by mAbs.

Homology analysis revealed that the five residues are highly conserved among PDCoV strains but share low sequence similarity (20%) with porcine alpha- and beta-CoVs (Fig. 4A and C), which suggested that the epitope is highly specific to PDCoV. Lau et al. (48) found a high similarity between the *S* genes of Munia-CoV and PDCoV, proposing that PDCoV might have emerged from a recombination event involving sparrow deltacoronavirus (SpDCoV) and Munia-CoV. We found that the five residues recognized by mAb 4A6 exhibited a 20% sequence identity with Munia-CoV and SpDCoV but displayed a 100% identity with ALCCoV and CFBCoV (Fig. 4B). Interestingly, only one amino acid residue (E465G) differed with thrush coronavirus (Thrush-CoV), and previously identified epitopes 280-FYSDPKSAV-288 and 506-TENNRFTT-513 in the PDCoV S protein showed complete alignment with ALCCoV and CFCCoV (36). These findings suggest that, apart from Munia-CoV and SpDCoV, other *deltacoronaviruses* may have played a role in the evolution of PDCoV, potentially facilitating its adaptation from avian hosts to mammalian hosts.

As shown in Fig. 5C through F, indirect ELISA and SPR results showed that mAb 4A6 had a similar affinity with the S1 protein of PDCoV isolates from China, the United States, and Thailand. The results of the plaque reduction neutralization test (PRNT) assay showed that mAb 4A6 had similar neutralizing activity against different PDCoV strains isolated from different pig-producing regions and from different years (Fig. 5G). This may be due to the mAb 4A6-recognized epitope being highly conserved among different PDCoV strains. The broad-spectrum neutralizing efficacy of mAb 4A6 against various PDCoV isolates indicates its potential to confer resilience against viral evolution. Consequently, mAb 4A6 could be strategically stockpiled for pandemic preparedness and utilized either as a standalone mAb therapy or in combination with other mAbs as a cocktail.

Considerable research has resulted in numerous mAbs that have been used successfully to treat SARS-CoV-2 (49, 50); these mAbs target the S protein. In addition, single-chain fragment variable antibodies, specifically PZZ 21, PZZ 24, and PZZ, have been assessed in pig models and have demonstrated potential efficacy in the prevention and treatment of PEDV infection (35). To date, there are no reports describing the clinical application of anti-PDCoV mAbs in pigs. Here, we evaluated the therapeutic efficacy of mAb 4A6 against PDCoV infection in Chenghua piglets. Notably, the Chenghua pig herds were consistently PDCoV seronegative, and Chenghua pigs were highly susceptible to PDCoV (data not show), so the Chenghua piglets were selected as the animal model in this study. Piglets were given either one or two doses of mAb 4A6 after PDCoV challenge (Fig. 6A). Compared with the control group, the two experimental groups exhibited a delayed onset of diarrhea and less severe symptoms. On the 4 dpc, two piglets in group E1, one piglet in group E2, and one piglet in group C1 died. This may be attributed to these piglets being smaller and weaker, making them more susceptible to PDCoV infection. qRT-PCR analysis of intestinal tissues revealed that despite the mortality of these four piglets, the viral RNA load was lower in the jejunum, ileum, and duodenum of the dead E1 and E2 group piglets than in the dead control group piglet. At the end of 7 days, we found no significant difference in viral RNA load in the jejunum and ileum of experimental and control piglets, but a statistically higher viral load in the duodenums of the control piglets compared to the experimental group 1 piglet (Fig. 7B).

The shedding of virus in feces was quantitated by qRT-PCR. mAb 4A6 therapy, either one or two doses, reduced peak viral loads compared to controls (Fig. 7A). HE staining results revealed a lower shedding of jejunal epithelial cells in piglets of the experimental groups compared to the control group (Fig. 7D). IHC staining results revealed significant staining in the jejunal epithelial cells of the control group piglets, while no obvious staining was observed in the jejunal epithelial cells of the experimental group piglets and all ileal epithelial cells of piglets (Fig. 7E). The efficacy of IHC staining is contingent upon the concentration of the antigen in tissue and the sensitivity of the antibody used (51). In Fig. 7B, the qRT-PCR results indicated that the viral load in the jejunum of the control group was not significantly different from that in groups E1 and E2; however, the viral load in the control group was slightly higher. In groups E1 and E2, it is likely that the viral load did not attain the requisite threshold for effective staining by anti-PDCoV N protein mAb 4E88. This insufficiency may therefore explain the lack of immunohistochemical staining of the viral antigen in the jejunal tissue observed in Fig. 7E for these groups.

In conclusion, mAb 4A6 treatment does have a therapeutic effect on PDCoV-infected piglets, although multiple treatments with mAb 4A6 may lead to improved outcomes. Also, mAb 4A6 is a mouse-derived antibody categorized as heterologous in pigs, resulting in a short half-life. The half-life is generally longer in chimeric antibodies (8–10 days) than in murine mAbs (2–3 days) (52). In our follow-up research, we plan to modify the murine antibody into a porcine-mouse chimeric antibody to reduce side effects and enhance therapeutic effects in piglets.

To our knowledge, this is the first evaluation of the therapeutic efficacy of a PDCoV-neutralizing antibody in piglets, our results show that administering mAb 4A6 can delay the onset of diarrhea symptoms, reduce the severity of diarrhea, and decrease virus shedding. Taken together, mAb 4A6 has promising therapeutic value for PDCoV-infected piglets, and the mAb 4A6 recognized epitope will also be useful for the development of epitope-based vaccine and antiviral drug of PDCoV.

## MATERIALS AND METHODS

### Cells, viruses, and mAbs

ST, HEK-293, and HEK-293T cells were cultured in Dulbecco’s Modified Eagle’s Medium (DMEM, Gibco, USA) supplemented with 10% FBS at 37°C in a humidified 5% CO_2_ atmosphere. The PDCoV strains CHN-SC2015, SCNC201705, and CHN-SCMY2021-01, propagated in ST cells, have been characterized in our laboratory. Their corresponding GenBank accession numbers are MK355396.1, MK572803.1, and OM047182.1, respectively. Monoclonal antibodies, 4A6 and 4E3, targeting the PDCoV S protein were generated in our laboratory. mAb 4A6 recognizes a conformational epitope within the first 552 amino acids of the S protein (hereafter referred to as S1). mAb 4E3 (36), used here as a positive control, recognizes the linear epitope 280-FYSDPKSAV-288 of S1 and effectively neutralizes PDCoV with an IC50 of 3.155 µg/mL. The mAbs were purified using protein G column chromatography (NUPTEC, Hangzhou, China) following the manufacturer’s instructions.

### Plasmid construction, protein expression, and purification

The S1 gene sequences of the different PDCoV strains CHN-SC2015, USA-Ohio137-2014, and Thailand-S5011-2015 were each cloned into into pcDNA 3.4(+) with a C-terminal human Fc tag, respectively. HEK 293 cells were transfected with the three plasmids using Lipofectamine 3000 (Invitrogen) according to the manufacturer’s protocol. After 48 h incubation, the culture medium was replaced with fresh medium containing 750 µg/mL geneticin (G418, Gibco, Thermo Fisher Scientific), and cells were maintained in this medium for 2 weeks. The HEK 293 cells stably expressing S1 proteins were harvested, and S1 proteins were purified as previously described with slight modifications (53). Briefly, the S1 proteins were purified using protein A/G magnetic beads following the manufacturer’s instructions. In addition, purified PDCoV S1-CTD (54) and N (41) proteins were stored in our laboratory.

### Identification of reactivity and neutralizing ability of mAb 4A6

To determine the reactivity of mAb 4A6 with PDCoV, an IFA assay was conducted as previously described (36). Briefly, ST cells were infected with PDCoV CHN-SC2015 (MOI = 1). At 36 h post-infection, cells were fixed with 4% paraformaldehyde for 30 min at room temperature, then permeabilized with 0.5% Triton-X-100 for 20 min at room temperature. Fixed cells were blocked with 2% bovine albumin V (BSA, Solarbio, China) in PBS for 30 min followed by 2 h incubation with 300 µL of 30 µg/mL purified mAb 4A6 or 4E3. Cells were washed then incubated for 1 h at 37°C with Alexa Fluor 555-labeled Donkey Anti-Mouse IgG antibody (1:200). Nuclei were stained with DAPI (Solarbio, Beijing, China).

To investigate the neutralization mechanism of mAb 4A6, pre- and post-attachment neutralization assays were done as previously reported (30). Briefly, for pre-attachment assays, twofold serially diluted mAb 4A6 was prepared at 4°C in DMEM and incubated with 100 TCID50 of PDCoV CHN-SC2015 for 1 h at 4°C. The mAb/PDCoV was added to ST cells and incubated for 1 h at 4°C. Then, the overlay of medium and an equal volume of 1.8% low melt agarose were added to each plate; plaques were counted 48–72 h post-infection. The percent neutralization was calculated as ([100 − number of plaques with antibodies]/number of plaques without antibodies) × 100%. For post-attachment assays, 100 TCID50 of PDCoV was inoculated onto ST cell cultures and incubated 1 h at 4°C. The supernatants were removed, and cells were washed twice with cold DMEM, followed by the addition of twofold serially diluted mAb 4A6 in cold DMEM. The plates were incubated for 1 h at 4°C, and the supernatants were then removed. Low melt agarose was added, and the neutralization rate was calculated as described above.

### Identification of the epitope recognized by mAb 4A6 via phage displays peptide library

To determine the epitope recognized by mAb 4A6, a Ph.D.-7 Phage Display Peptide Library (New England Biolabs, MA, USA), containing random 7-mer peptides, was screened with mAb 4A6 according to the manufacturer’s instructions. After three rounds of biopanning, 15 positive phage clones were randomly selected, and their reactivity to 4A6 was determined by sandwich ELISA. In brief, a 96-well plate coated with 100 µg/mL mAb 4A6 (100 µL/well) was incubated with 100 µL of fourfold serially diluted positive phage, then with 100 µL HRP-conjugated anti-M13 antibody (Sino Biological, Inc., Beijing, China). The single-stranded phage DNAs from the verified phage clones were extracted and sequenced with the 96 gIII sequencing primer (5′-TGAGCGGATA ACAATTTCAC-3′). The phage peptide sequences were deduced from the DNA sequences and aligned with PDCoV *S* gene sequence using *DNAMAN* software.

### Immunization of mice

To prepare specific anti-mimotope sera, a mimotope P1 peptide (QYPVSYA) was synthesized and coupled to KLH. The conjugate, called KLH-P1, was confirmed using mass spectrometry and high-performance liquid chromatography (HPLC). Female BALB/c mice (6 weeks old) were obtained from Chengdu Dossy Experimental Animal Co, Ltd and divided into 2 groups of 5 mice/group. The experimental group was subcutaneously injected with KLH-P1 (50 µg/mouse), and the negative control group was injected with the same volume of sterile PBS. All animals were boosted at 2-week intervals. Serum samples were collected weekly for 6 weeks. All sera were heat-inactivated for 1 h at 56°C and stored at −20°C. Mouse serum IgG titers and neutralizing antibody titers were assessed as previously described (36).

### Identification of the essential amino acids recognized by mAb 4A6

The structure of S1-CTD was modeled using SWISS-MODEL (https://swissmodel.expasy.org/), and the variable region of mAb 4A6 was modeled using the Discovery Studio 2019 software. Protein-protein docking simulations were done to generate well-fitting conformations of mAb 4A6 to potential binding pockets of S1-CTD. Polar hydrogens and partial charges were added to the protein structures without hetero atom using MOE software. The conformations with the lowest binding energy were analyzed in 3D using PyMOL, and the best models were chosen for further study.

To determine the essential amino acids in the S1 protein needed for mAb 4A6 binding, a series of point mutations were made in the binding region of the PDCoV S1-CTD protein. The WT S1 sequence and the mutations S1-S461A, S1-P462A, S1-T463A, S1-E465A, and S1-Y467A were cloned into a pcDNA3.4 (+) vector with a C-terminal human Fc tag. HEK-293T cells were transfected with these constructs for 36 h, then harvested and lysed in a cold lysis buffer (Beyotime). The proteins were then purified using Protein A + G magnetic beads (Beyotime, Shanghai, China). The IFA conducted as described above and the dot blot assay was conducted as previously described (41).

### Homology analysis

To determine the conservation level of the essential amino acid residues (S461, P462, T463, E465, and Y467) involved in mAb 4A6 binding, 25 PDCoV strains from nine countries were selected for sequence alignment and compared using *MEGA-X* and *DNASTAR* software. The conservation level of these amino acids among other delta-CoVs and porcine CoV was also determined.

### Assessment of the broad-spectrum efficacy of 4A6 against various PDCoV strains

Indirect ELISA was used to determine the reactivity of mAb 4A6 with the S1 protein of different PDCoV strains. Briefly, 96-well plates were coated with purified PDCoV S1 proteins then blocked with 5% skim milk in PBST for 2 h at 37°C. One hundred microliter of 10-fold serially diluted purified mAbs (from 100 μg/mL to 0.001 μg/mL) were aliquoted into wells and incubated for 1 h at 37°C. After washing again, 100 µL of HRP conjugated anti-mouse IgG (1:5,000) was aliquoted into wells and incubated for 30 min at 37°C. After washing four times with PBST, antibody binding was analyzed by the addition of TMB solution (Solarbio, China); reactions were stopped by addition of 2 M H_2_SO_4_. The optical density at 450 nm was read on a microplate absorbance reader (Bio-Rad, USA), and EC50 was determined.

The binding kinetics and affinity of mAb 4A6 with S1 protein of different PDCoV strains was evaluated by SPR as previously described (55). SPR experiments were performed using a BIACORE T-200 (GE Healthcare) equipped with a Series CM5 sensor chip. The S1 protein of three different PDCoV strains was immobilized using amine-coupling chemistry as indicated in the BIACORE T-200 wizard program. Three S1 proteins at a concentration of 18 µg/mL in 10  mM sodium acetate, pH 5.0, were immobilized at a density of 370 resonance units (RU). Subsequently, blocking was performed with ethanolamine. To analyze the binding affinity of mAb 4A6, it was injected over the two flow cells at concentrations ranging from 1.5625 to 200 nM, with a flow rate of 30 µL/min. The *Biacore T-200* evaluation software (GE Healthcare) was used for data processing, and the KD value was calculated by affinity fitting.

To assess the broad-spectrum neutralizing activity of the mAb 4A6 against various strains of PDCoV, a PRNT was conducted with three different PDCoV strains: CHN-SC2015, SCNC201705, and CHN-SCMY2021-01, which were isolated in different years and different pig-producing region. PRNT was performed as previously described (36).

### Therapeutic efficacy of mAb 4A6 in PDCoV-challenged pigs

Fifteen seven-day old Chenghua piglets were randomly divided into three groups (experimental groups 1 and 2 and the control group; referred to hereafter as E1−1 ~ E1−5, E2−1 ~ E2−5, and C1−C5, respectively). All piglets were screened for the absence of PDCoV antibody by indirect-ELISA and orally challenged with 2 mL of inoculum containing 2 × 10^7^ tissue culture infectious dose (TCID50) PDCoV. Twenty-four hours post-challenge groups E1 and E2 received, by intramuscular injection, 3 mg/kg of mAb 4A6. Control group was injected with same volume of PBS. Forty-eight hours post-challenge group E1 again received 3 mg/kg mAb 4A6. At 7 dpc, the remaining piglets were euthanized and necropsied. Intestinal tissues were collected for quantitative analysis of viral load, and jejunum and ileum sections were stained with hematoxylin and eosin. PDCoV-specific antigen was detected by IHC staining using anti-PDCoV N protein mAb 4E88. The detailed procedure has been previously described (56).

### Statistical analysis

All experimental data were analyzed using GraphPad Prism version 8.0 and expressed as mean ± SD. Differences among the groups were analyzed by one-way ANOVA and two-way ANOVA as appropriate. Statistical significance is indicated by * *P* value < 0.05, ** *P* value < 0.01, *** *P* value < 0.001, and ****P value < 0.0001.

## Data Availability

The data that support the findings of this study are available on request from the corresponding author.
